# 18F-FDG PET/MRI and 18F-FDG PET/CT for the Management of Gynecological Malignancies: A Comprehensive Review of the Literature

**DOI:** 10.3390/jimaging9100223

**Published:** 2023-10-13

**Authors:** Leila Allahqoli, Sevil Hakimi, Antonio Simone Laganà, Zohre Momenimovahed, Afrooz Mazidimoradi, Azam Rahmani, Arezoo Fallahi, Hamid Salehiniya, Mohammad Matin Ghiasvand, Ibrahim Alkatout

**Affiliations:** 1 Ministry of Health and Medical Education, Tehran 1467664961, Iran; 2 Faculty of Nursing and Midwifery, Research Center of Psychiatry and Behavioral Sciences, Tabriz University of Medical Sciences, Tabriz 516615731, Iran; hakimisevil@gmail.com; 3 Unit of Obstetrics and Gynecology, “Paolo Giaccone” Hospital, Department of Health Promotion, Mother and Child Care, Internal Medicine and Medical Specialties (PROMISE), University of Palermo, 90127 Palermo, Italy; antoniosimone.lagana@unipa.it; 4 Department of Midwifery and Reproductive Health, Qom University of Medical Sciences, Qom 3716993456, Iran; momeniz@gmail.com; 5 Neyriz Public Health Clinic, Shiraz University of Medical Sciences, Shiraz 7134845794, Iran; mazidimoradi.8836@gmail.com; 6 Nursing and Midwifery Care Research Center, School of Nursing and Midwifery, Tehran University of Medical Sciences, Tehran 141973317, Iran; arahmani@sin.tums.ac.ir; 7 Department of Public Health, Faculty of Health, Kurdistan University of Medical Sciences, Sanandaj 6617713446, Iran; arezofalahi91@gmail.com; 8 Social Determinants of Health Research Center, Birjand University of Medical Sciences, Birjand 9717853076, Iran; alesaleh70@yahoo.com; 9 Department of Computer Engineering, Amirkabir University of Technology (AUT), Tehran 1591634311, Iran; moh.gh@aut.ac.ir; 10 University Hospitals Schleswig-Holstein, Campus Kiel, Kiel School of Gynaecological Endoscopy, Arnold-Heller-Str. 3, Haus 24, 24105 Kiel, Germany; ibrahim.alkatout@uksh.de

**Keywords:** imaging, 18F-FDG PET/CT, 18F-FDG PET/MRI, hybrid FDG PET/MRI, gynecologic malignancies, ovarian cancer, endometrial cancer, cervical cancer, vulvar and vagina cancer

## Abstract

Objective: Positron emission tomography with 2-deoxy-2-[fluorine-18] fluoro- D-glucose integrated with computed tomography (18F-FDG PET/CT) or magnetic resonance imaging (18F-FDG PET/MRI) has emerged as a promising tool for managing various types of cancer. This review study was conducted to investigate the role of 18F- FDG PET/CT and FDG PET/MRI in the management of gynecological malignancies. Search strategy: We searched for relevant articles in the three databases PubMed/MEDLINE, Scopus, and Web of Science. Selection criteria: All studies reporting data on the FDG PET/CT and FDG PET MRI in the management of gynecological cancer, performed anywhere in the world and published exclusively in the English language, were included in the present study. Data collection and analysis: We used the EndNote software (EndNote X8.1, Thomson Reuters) to list the studies and screen them on the basis of the inclusion criteria. Data, including first author, publication year, sample size, clinical application, imaging type, and main result, were extracted and tabulated in Excel. The sensitivity, specificity, and diagnostic accuracy of the modalities were extracted and summarized. Main results: After screening 988 records, 166 studies published between 2004 and 2022 were included, covering various methodologies. Studies were divided into the following five categories: the role of FDG PET/CT and FDG-PET/MRI in the management of: (a) endometrial cancer (*n* = 30); (b) ovarian cancer (*n* = 60); (c) cervical cancer (*n* = 50); (d) vulvar and vagina cancers (*n* = 12); and (e) gynecological cancers (*n* = 14). Conclusions: FDG PET/CT and FDG PET/MRI have demonstrated potential as non-invasive imaging tools for enhancing the management of gynecological malignancies. Nevertheless, certain associated challenges warrant attention.

## 1. Introduction

Gynecological malignancies, prevalent cancers affecting women globally, result in substantial morbidity and mortality [1,2,3]. Effective management and improved survival rates necessitate early diagnosis, accurate staging, and the prompt recurrence detection [4].

Various imaging modalities, including ultrasound, computed tomography (CT), magnetic resonance imaging (MRI), functional MRI, and combined imaging with positron emission tomography (PET/MRI) and positron-emission-tomography-computed tomography (PET-CT), are employed for the management of common gynecologic malignancies, specifically cervical, endometrial, and ovarian malignancies [4]. Recent advancements in imaging, such as 18F-fluorodeoxyglucose positron-emission-computed tomography (18F-FDG PET/CT) and 18F-FDG-PET/MRI, have significantly contributed to the characterization and management of gynecological malignancies [5]. While the use of FDG PET/CT for gynecological cancer management is not mandatory, it has been recommended in the Danish Gynecological Cancer Ovarian Guidelines since 2009 for evaluating women with suspected ovarian cancer. FDG PET/CT aids in cancer diagnosis, metastatic lymph node detection, and identifying distant metastases [6]. 

Upon reviewing the literature, we identified conflicting data regarding the role of FDG PET/CT and FDG PET/MRI in gynecological cancers. These hybrid imaging techniques, in conjunction with robotic-assisted surgical tools and artificial intelligence (AI) applications for personalized treatment strategies, hold promise for achieving improved outcomes with fewer adverse effects. Thus, believing that emerging imaging technologies will play a pivotal role in gynecological cancer diagnosis and treatment, this study was designed to assess the proposed roles of FDG PET/CT and FDG PET/MRI in managing various types of gynecological cancers.

## 2. Materials and Methods 

### 2.1. Search Strategy and Information Sources

We conducted a thorough search for pertinent articles in three databases: PubMed/MEDLINE, Scopus, and Web of Science. The following keywords were used for the search in March 2023: “gynecologic malignancies”, “gynecological neoplasms”, “gynecological tumors”, “management”, “hybrid imaging using 18F-FDG PET CT” and “18F- FDG PET/MRI”. MeSH keywords and Boolean (AND, OR) operators were employed to enhance the selection of entries.

### 2.2. Study Selection

We utilized EndNote software (EndNote X8.1 BLD 11010, Thomson Reuters) to compile and screen studies based on inclusion criteria. The study selection process consisted of three phases: screening, selection, and data extraction. During the screening phase, two trained authors (LA and AMM) evaluated titles and abstracts of all retrieved articles. Of these, 204 articles were selected for full-text review. Two authors (LA and HS) independently completed a checklist-style form and included articles meeting the inclusion criteria. Any discrepancies during the full-text review were resolved by a third expert (IA). We included all studies reporting data on FDG PET/CT and FDG PET/MRI in gynecological cancer management, published exclusively in English, without imposing a time limit on the search. Exclusions comprised review studies, in vitro or animal studies, studies related to cancer during pregnancy, and studies employing mathematical, deep learning, or radiomics models to assess the two imaging methods or quantitative parameters, like maximum standardized uptake value (SUVmax), metabolic tumor volume (MTV), and total lesion glycolysis (TLG).

### 2.3. Data Extraction and Synthesis

We extracted data from the included studies using a standardized form and recorded it in Excel. Extracted information included the first author, publication year, sample size, clinical application, imaging modality, and main result. Any disagreements were resolved through discussion, involving a third external colleague when necessary. Given the diversity in reporting methods, we conducted a narrative synthesis of the studies and summarized sensitivity, specificity, and diagnostic accuracy data.

## 3. Results

### 3.1. Search Results

**Figure 1:**
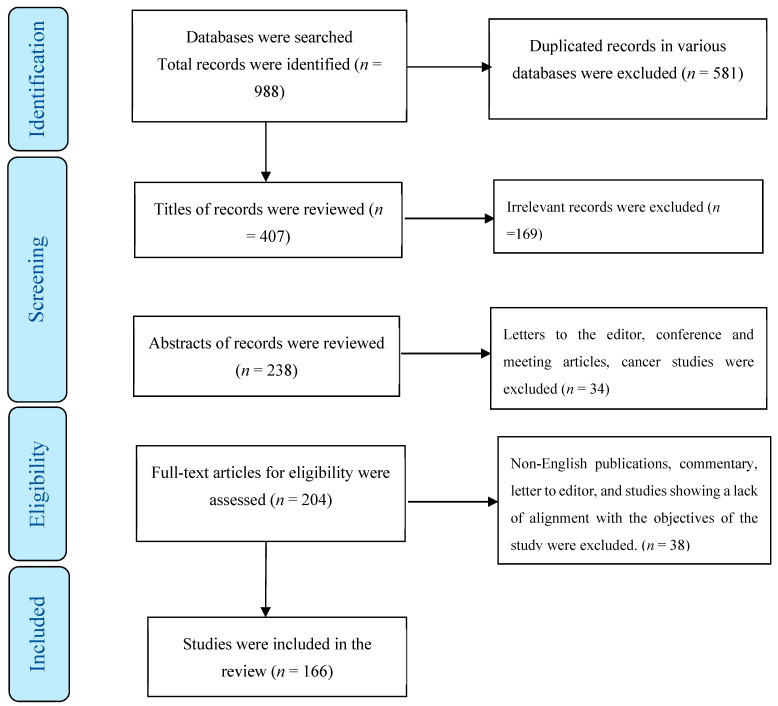
The process of screening and selecting relevant studies.

In total, 988 publications were identified across various databases, of which 581 were duplicates. After evaluating the titles and abstracts of the remaining papers, 203 were excluded. Of the remaining articles, 38 were omitted due to a lack of alignment with the study objectives. Ultimately, the present review encompassed 166 studies (Figure 1) employing various methodological approaches.

### 3.2. Study Characteristics

One hundred and sixty-six studies met the criteria for inclusion in this current review. These studies were categorized into five groups as follows: (a) FDG PET/MRI and FDG PET/CT for the management of endometrial cancer (EC) (*n* = 30), (b) ovarian cancer (OC) (*n* = 60), (c) cervical cancer (CC) (*n* = 50), (d) vulvar and vagina cancers (*n* = 12), and (e) gynecological cancers (*n* = 14). Studies that did not report separate results for gynecological cancers were included in the fifth group. 

#### 3.2.1. FDG PET/CT and FDG PET/MRI in the Management of the EC

We identified 30 studies that examined the role of FDG PET/CT and FDG PET/MRI in managing EC. These studies were further categorized into the following subgroups: (a) the staging or diagnosis of primary EC (*n* = 9), (b) the diagnosis or prediction of recurrence of EC (*n* = 5), and (c) the detection or prediction of metastases of EC (*n* = 16). 

##### Staging or Diagnosis of Primary EC

Five studies, involving a total of 506 patients, focused on staging EC using FDG PET/CT or FDG PET/MRI [7,8,9,10,11]. The sensitivity and specificity of FDG PET/CT for EC staging ranged from 90% to 93% and 49% to 96.3%, respectively [7,9]. In contrast, FDG PET/MRI exhibited a sensitivity and specificity of 77% and 84%, respectively [8]. The overall accuracy for staging was 86.0% for PET/MRI and 77.2% for PET/CT [11]. Additionally, four eligible FDG PET/CT studies, involving 158 patients, addressed the diagnosis of primary EC [12,13,14,15]. The sensitivity and specificity of FDG PET/CT for EC diagnosis ranged from 90.6% to 98.1% and 33.3% to 99%, respectively [12,14,15]. The overall accuracy of EC diagnosis with FDG PET/CT was 94.6% [12]. Bezzi and colleagues conducted a systematic review and meta-analysis on the use of hybrid FDG PET/MR imaging for EC staging/re-staging, analyzing eleven articles. Their results indicated that FDG PET/MRI is a valid imaging technique for EC patients, both for staging and re-staging, while also potentially reducing radiation exposure [16]. However, the limited availability in the literature of studies on FDG PET/MRI underscores the need for more prospective trials with larger and more homogeneous cohorts. 

##### Diagnosis or Prediction of Recurrence of EC

In this study, we evaluated the diagnostic accuracy of FDG PET/CT in cases of recurrent EC. Five eligible FDG PET/CT studies, involving 345 patients, were included in the assessment of recurrent EC diagnosis [17,18,19,20,21]. The sensitivity and specificity of FDG PET/CT in studies were reported as being greater than 88.9% and 93.6%, respectively. The overall accuracy for EC diagnosis with FDG PET/CT ranged from 91% to 97% [18,19,20,21]. In a systematic review by Bollineni et al. in 2016, F-FDG PET/CT exhibited excellent diagnostic performance for detecting postoperative recurrence in EC patients [22].

##### Detection or Prediction of Metastases of EC

The diagnostic value of FDG PET/CT and FDG PET/MRI in detecting EC metastases was assessed through 15 studies involving 1904 patients [23,24,25,26,27,28,29,30,31,32,33,34,35,36,37,38]. The sensitivity and specificity for FDG PET/CT ranged from 45.4% to 100% and 66.67% to 99.8%, respectively [23,24,25,26,27,28,29,30,31,32,33]. In contrast, FDG PET/MRI demonstrated a sensitivity of 100% and specificity of 96.9% for detecting regional nodal metastasis [38]. The range of overall accuracy of diagnosis of EC for FDG PET/MRI was 81.8–97% [36,38].

A 2016 systematic review and meta-analysis by Bollineni et al. aimed to investigate the diagnostic performance of (18)F-FDG PET/CT in the preoperative evaluation of endometrial cancer recurrence (ECR) and lymph node metastases (LNM) in EC patients. Their analysis included 21 studies. The pooled sensitivity, specificity, positive likelihood ratio (LH+), negative likelihood ratio (LH-), diagnostic odds ratio (DOR), and area under the ROC curve (AUC) for F-FDG PET/CT in detecting ECR were 0.95, 0.91, 8.8, 0.08, 171.7, and 0.97, respectively. For the diagnosis of LNM, these values were 0.72, 0.94, 10.9, 0.36, 39.7, and 0.94, respectively [22]. In a 2021 study by Bezzi et al., PET/MRI was found to offer an opportunity to distinguish post-treatment changes from local recurrence and to detect small and indeterminate lymph node metastases in patients with recurrent EC, showing superior performance compared to other imaging modalities [16]. Based on the literature review, it can be concluded that FDG PET/CT exhibits appropriate diagnostic performance for the preoperative detection of LNM in EC patients [22]. 

Metastasis contributes to the vast majority of cancer-related mortality and the regulatory mechanisms of the multistep invasion–metastasis cascade are gradually being uncovered. The tumor suppressor p53 is the most frequently mutated gene in human cancers. Accumulating evidence suggests that TP53 mutations not only result in loss-of-function or dominant negative effects but also promote gain-of-function characteristics. Specifically, gain-of-function mutant p53 has been implicated in enhancing cancer cell motility, invasion, and metastasis [39]. The molecular classification of EC has independent prognostic value, particularly among women with high-grade EC. P53-aberrant EC is associated with poor clinical outcomes [40]. However, it remains unclear whether PET/CT or PET/MRI can further predict distant metastasis in patients with p53 mutations, and this issue warrants further investigation in future studies.

The roles of PET/CT and PET/MRI in the management of the EC are summarized in Table 1. 

#### 3.2.2. FDG PET/CT and FDG PET/MRI in the Management of OC

We identified 60 studies that examined the utilization of FDG PET/MRI and FDG PET/CT in the management of ovarian cancer (OC). These studies were categorized into the following subgroups: (a) the staging and diagnosis performance of OC (*n* = 12), (b) the prediction of the optimal primary treatment/treatment prognosis and response of OC (*n* = 6), (c) the diagnosis or prediction of recurrence of OC (*n* = 31), (d) the detection or prediction of metastases of OC (*n* = 9), and (e) the prediction of OC survival (*n* = 2). 

##### Staging and Diagnosis Performance of OC

We reviewed three FDG PET/CT studies involving a total of 102 patients aimed at staging OC [41,42,43]. The sensitivity and specificity of the pre-surgical staging of OC was 78% and 68%, respectively [43]. Additionally, FDG PET/CT and FDG PET/MRI demonstrated accuracy rates of 71% and 92.5%, respectively, in the peritoneal staging and characterization of suspected OC [41]. FDG PET/MRI exhibited an accuracy of 92.5% for characterizing suspected OC [44]. Moreover, eight eligible FDG PET/CT studies, with a total of 766 patients, addressed the diagnosis of OC. The sensitivity and specificity of FDG PET/CT for OC diagnosis ranged from 82.4% to 94.7% and 76.9% to 100%, respectively [45,46,47,48,49,50,51,52]. FDG PET/CT showed an accuracy of 81.1% to 92.9% for detecting primary OC [46,48,49,51]. In a systematic review by Suppiah and et al., 23 studies regarding the role of PET/CT in the diagnosis (10 articles, 734 patients) and staging (13 studies, 604 patients) were reviewed and the range of sensitivity and specificity of PET/CT in the diagnosis of OC was 82.4–100%% and 33.0–78.9%, respectively [53]. Although CT remains the preferred imaging modality for assessing ovarian cancer, PET/CT appears advantageous for staging and follow-up in OC [54].

##### Prediction Optimal Primary Treatment/Treatment Prognosis and Response of OC

We assessed two FDG PET/CT studies involving a total of 75 patients that aimed to predict optimal primary treatment in OC patients [55,56]. These studies reported sensitivity, specificity, and accuracy values of 91%, 67%, and 86%, respectively, for characterizing ovarian masses with PET/CT [55]. Additionally, results from four FDG PET/CT studies, including 134 patients, focused on assessing treatment prognosis and response in OC [57,58,59,60]. These studies demonstrated that FDG PET/CT is valuable for evaluating neoadjuvant chemotherapy response [57,59,60]. 

##### Diagnosis or Prediction of Recurrence of OC

In the present study, we evaluated the diagnostic accuracy of FDG PET/CT for recurrent OC. We reviewed 30 FDG PET/CT studies, which reported sensitivity and specificity values ranging from 40.7% to 100% and 55.5% to 100%, respectively. FDG PET/CT exhibited an accuracy of 72% to 97% for detecting recurrent OC [61,62,63,64,65,66,67,68,69,70,71,72,73,74,75,76,77,78,79,80,81,82,83,84,85,86,87,88,89,90,91]. A meta-analysis conducted in 2013 estimated the diagnostic accuracy of PET/CT for suspected recurrent OC by reviewing 29 studies (1651 patients with OC). The pooled sensitivity, specificity, positive likelihood ratio (LH+), negative likelihood ratio (LH-), and diagnostic odds ratio (DOR) for recurrent OC were 88.6%, 90.3%, 6.104, 0.122, and 57.032, respectively [92]. These findings suggest that FDG PET/CT is a valuable tool for predicting the diagnosis and restaging of suspected recurrent OC. 

##### Detection or Prediction of Metastases of OC

Nine FDG PET/CT studies involving 746 patients focused on diagnosing metastases in OC [42,93,94,95,96,97,98,99,100]. These studies reported sensitivity and specificity values of 64% to 96.2% and 90% to 98.2%, respectively, for FDG PET/CT in diagnosing peritoneal carcinomatosis, distant metastases, and detecting nodal metastases [42,94,95,96,98]. FDG PET/CT showed an accuracy of 88.6% to 95.6% for diagnosing OC metastasis [94,96]. A systematic review conducted in 2012 compared the diagnostic performance of PET/CT, CT, and MR for detecting metastatic lymph nodes in OC patients. This review, encompassing 18 relevant studies with a total of 882 patients, revealed that PET/CT was more accurate, with a sensitivity, specificity, and odds ratio (OR) of 73.2%, 96.7%, and 90.32, respectively [101]. 

A meta-analysis in 2022 reported that the sensitivity, specificity, and area under the ROC curve (AUC) of FDG PET/CT for diagnosing epithelial OC recurrence were 0.88, 0.89, and 0.94, respectively [102]. Collectively, these results suggest that FDG-PET or FDG PET/CT is more accurate than CT and MR imaging for detecting lymph node metastasis in OC patients, although its superiority for intra-abdominal disease spread remains debatable, while, in a study by Hynninen et al., PET/CT was not superior to CT for the detection of intra-abdominal disease spread but was more effective for the detection of extra-abdominal disease than CT [42]. 

##### Prediction of OC Survival

The role of FDG PET/CT in the prediction of OC survival was evaluated in the present study. We found that a negative FDG PET/CT has a high negative predictive value for disease presence and is notably associated with a very good disease-specific survival outcome [103,104]. The roles of FDG PET/CT and FDG-PET/MRI in the management of the OC are summarized in Table 2. 

#### 3.2.3. FDG PET/CT and FDG PET/MRI in the Management of CC

We encountered 50 studies that have delved into the utilization of FDG PET/CT and FDG PET/MRI for managing cervical cancer (CC). These studies were categorized into the following groups: (a) the staging or diagnosis of primary CC (*n* = 12), (b) the treatment prognosis and prediction of response to treatment of CC (*n* = 8), (c) the diagnosis or prediction of recurrence of CC (*n* = 3), (d) the detection or prediction of CC metastasis (*n* = 21), and (e) the prognostic value and prediction of survival of CC (*n* = 6). 

##### Staging or Diagnosis of Primary CC

In the present study, we evaluated the diagnostic accuracy of FDG PET/CT versus FDG PET/MRI in staging and diagnosing CC. A total of eight FDG PET/CT or PET/MRI studies focusing on CC staging were reviewed [106,107,108,109,110,111,112,113]. The study by Anner et al. in 2018 found that PET/MRI exhibited slightly superior results compared to PET/CT in terms of specificity (77% vs. 69%), positive predictive value (PPV) (75% vs. 69%), and negative predictive value (NPV) (67% vs. 64%) [106]. Conversely, Lazzari et al.’s 2014 study favored FDG PET/CT for CC staging and disease definition [109]. The sensitivity and specificity of FDG PET/MRI in CC staging across studies ranged from 77% to 91% and 90% to 94% [106,110,111].

For diagnosing CC, five eligible studies utilizing FDG PET/CT and FDG PET/MRI with a total of 188 patients were included [114,115,116,117,118]. The sensitivity and specificity of FDG PET/CT in CC diagnosis ranged from 75% to 86.66% and 44.4% to 96%, respectively [115,118]. The diagnostic accuracy of PET/CT for early-stage CC was reported as 95.45% [117]. In contrast, the accuracy, sensitivity, and NPV of FDG PET/MRI in CC diagnosis were 78.5%, 64.9%, and 74.5%, respectively [116]. Given that the present study is the first to address the diagnostic accuracy of FDG PET/CT versus FDG PET/MRI in CC staging and diagnosis, direct data comparison is not feasible. MRI has higher imaging sensitivity in detecting the area in which the cervical cancer involved, while PET imaging can provide the favorable detection of cervical cancer [119]. It is unknown whether MRI, with its superior soft-tissue contrast, can improve the combined accuracy of a PET/MRI scanner as compared to PET/CT in detecting malignant lymph nodes [120] or whether it is useful in image-guided adaptive brachytherapy [121]. Further in-depth studies are recommended to explore this issue comprehensively.

##### Treatment Prognosis and Prediction of Response to Treatment of CC

Eight eligible FDG PET/CT studies involving 728 patients were analyzed to assess the role of FDG PET/CT in treatment prognosis and in predicting the response to CC treatment [122,123,124,125,126,127,128,129]. Draghini et al.’s 2019 study found that FDG PET/CT significantly influenced stage assessment and radiotherapy treatment planning due to its high specificity in detecting distant metastases and nodal involvement [128]. The sensitivity and specificity of PET/CT in detecting or predicting residual local and regional disease ranged from 20% to 94.8% and 62% to 100% [122,129,130]. The accuracy of PET/CT for detecting residual local and regional disease was reported as 89% [130]. In a 2012 study by Chang et al., post-treatment PET/CT scans were deemed sensitive and accurate for monitoring and providing prognostic information in CC [129]. A systematic review in 2021 assessed the diagnostic accuracy of FDG PET/CT in predicting tumor response in chemotherapy-treated advanced cervical cancer (LACC). This review, covering 15 articles with 1132 patients published from 2010 to 2020, reported sensitivities and specificities of PET/CT at 83.5% and 77.8%, respectively. The diagnostic sensitivity and specificity of PET/CT for detecting residual tumor were 86% and 95%, respectively [131]. While FDG PET/CT shows promise in assessing treatment response, further studies are needed to establish it as a standard option for evaluating treatment response. 

##### Diagnosis or Prediction of Recurrence of CC

In our study, we included three eligible FDG PET/CT studies with a total of 74 patients focused on diagnosing recurrence [132,133,134]. The sensitivity, specificity, and accuracy of PET/CT for detecting recurrent CC were 90.3%, 81.0%, and 86.5%, respectively [134]. A systematic review and meta-analysis conducted by Chu et al. in 2014 assessed the diagnostic value of PET or FDG PET-CT in recurrent CC. Twenty studies were included, and the meta-analysis indicated pooled sensitivity and specificity values of 0.82 and 0.98, respectively, for local-regional recurrence [135]. In summary, FDG-PET has demonstrated its value in assessing recurrent CC.

##### Detection or Prediction of CC Metastasis

This study examined the diagnostic value of FDG PET/CT in detecting pelvic lymph nodal and para-aortic nodal metastasis of CC through 21 eligible FDG PET/CT studies comprising 1729 CC patients [136,137,138,139,140,141,142,143,144,145,146,147,148,149,150,151,152,153,154,155,156]. The sensitivity and specificity of FDG PET/CT in detecting CC metastasis ranged from 28.6% to 92.8% and 58.33% to 98.8%, respectively [136,137,138,139,140,141,142,143,144,145,146,147,148,150,152,153,154,155,156]. The accuracy of PET/CT in detecting CC metastasis was reported to range from 65.1% to 99.3% [136,142,143,144,148,150]. Adam and co-workers conducted a systematic review evaluating FDG-PET/CT’s performance for lymph node metastases in LACC, reviewing twelve studies with 778 patients. For pelvic nodes, summary estimates of sensitivity, specificity, LR+, and LR- were 0.88, 0.93, 11.90, and 0.13, respectively [157]. It is important to note that PET/CT plays a valuable role in guiding radiation field planning for locally advanced cervical cancer. PET/CT can effectively detect pelvic nodes and guide clinicians in making decisions regarding radiation fields to eliminate potential microscopic para-aortic metastases [158,159].

##### Prognostic Value and Prediction of Survival of CC

We explored the prognostic value of FDG PET/CT in CC through six studies involving 571 patients [160,161,162,163,164,165]. The estimated 5-year overall survival (OS), disease-free survival (DFS), and local control (LC) rates ranged from 81% to 82%, 70% to 74%, and 84.4% to 86%, respectively [162,165]. The extent of lymph node involvement in para-aortic, retrocrural, and supraclavicular areas was identified as a significant prognostic factor for CC progression [160,161,163]. A systematic review by Han et al. in 2018 analyzed twelve studies comprising 660 patients and indicated that volume-based FDG PET/CT parameters were significant prognostic factors in CC patients [166]. In summary, FDG PET/CT stands out as the preferred method for staging advanced CC. In addition, in locally advanced CC, FDG PET/CT may be used for staging, in planning for radiotherapy, and in evaluating treatment response, disease recurrence, and long-term survival [167]. The role of FDG PET/CT and FDG-PET/MRI for the management of CC is summarized in Table 3.

#### 3.2.4. FDG PET/CT in the Management of Vulvar and Vagina Cancers

We identified 12 studies that focused on the use of FDG PET/CT in the management of vulvar and vaginal cancers. These studies were categorized into the following groups: (a) the staging or diagnosis performance of vulvar and vaginal cancer (*n* = 8), (b) the recurrence of vulvar (*n* = 1), and (c) the detection and prediction of metastases of the vulvar and vaginal cancer (*n* = 3).

##### Staging or Diagnosis Performance of Vulvar and Vaginal Cancer

In our current investigation, we evaluated the diagnostic accuracy of FDG PET/CT for staging and diagnosing vulvar and vaginal cancers [168,169,170,171,172,173,174,175]. A total of four FDG PET/CT studies involving 243 patients, which aimed to stage vulvar cancer, were examined. The range of sensitivity, specificity, and accuracy of FDG PET/CT for vulvar staging was found to be 50–100%, 65.5–89%, and 59–82.5%, respectively [168,169,170,171]. Additionally, four eligible FDG PET/CT studies with a total of 79 patients were included in the diagnosis of vulvar and vaginal cancers [172,173,174,175]. FDG PET/CT exhibited a sensitivity of 95% and a PPV of 100% in identifying the primary tumor [173]. In the study by Peiro et al., PET/CT demonstrated a sensitivity of 100% for detecting vulvar lesions in squamous cell carcinomas and 60% in non-squamous cell carcinomas [174]. A systematic review conducted by Triumbari et al. in 2021 assessed the role of FDG PET/CT in vulvar cancer patients and provided summary estimates of its diagnostic performance for preoperative lymph node staging. The review of ten articles revealed sensitivity, specificity, PPV, NPV, and DOR values of 0.70, 0.90, 0.86, 0.77, and 10.49, respectively [176].

##### Recurrence of Vulvar Cancer

In our study, we also investigated the role of FDG PET/CT in detecting recurrences of vulvar cancer. In a retrospective multicentric study, PET/CT showed sensitivity, specificity, PPV, NPV, and accuracy values of 100%, 92%, 98%, 100%, and 98%, respectively. FDG PET/CT proved to be a precise tool for assessing recurrent vulvar cancer with high sensitivity and specificity, significantly impacting clinical decision-making [177]. However, due to the limited number of additional studies available, a direct data comparison was not feasible.

##### Detection and Prediction of Metastases Vulvar and Vaginal Cancer

The diagnostic value of FDG PET/CT in detecting and predicting metastases in vulvar and vaginal cancer was another aspect examined in our study. A total of three FDG PET/CT studies involving 180 patients, aimed at detecting and predicting metastases in vulvar cancer, were reviewed [178,179,180]. The range of sensitivity, specificity, and accuracy of FDG PET/CT in detecting metastases in vulvar and vaginal cancer was found to be 56–89%, 67–88%, and 74–84%, respectively [178,179]. In summary, our study delved into the various aspects of FDG PET/CT’s role in managing vulvar and vaginal cancers, including the staging, diagnosing, assessing of recurrences, and detecting of metastases. These findings shed light on the potential of FDG PET/CT as a valuable tool in the management of these cancers, although further research is needed to expand our understanding of its full capabilities in these domains.

Three domains of FDG PET/CT and FDG-PET/MRI in the management of vulvar and vaginal cancer are summarized in Table 4.

#### 3.2.5. FDG PET/MRI and FDG PET/CT in the Management of Gynecological Cancer

We identified 15 studies that investigated the use of FDG PET/MRI and FDG PET/CT in the management of gynecological cancers. These studies were categorized into the following groups: (a) the diagnosis of primary or recurrent gynecological cancers (*n* = 10) and (b) the detection and prediction of metastases of gynecological cancers (*n* = 4).

##### Diagnosis of Primary or Recurrent Gynecological Cancers

We reviewed a total of nine FDG PET/CT and FDG-PET/MRI studies focused on diagnosing gynecological cancers [181,182,183,184,185,186,187,188,189,190]. The sensitivity and specificity of FDG PET/CT for detecting malignant or borderline malignant pelvic tumors ranged from 50% to 71.4% and 81.3% to 93.9%, respectively [183,184]. Notably, Sawicki et al. found that FDG-PET/MRI correctly identified cancer recurrence in 100% of patients with a diagnostic accuracy of 99.2% [186]. Additionally, Schwartz et al. (2018) suggested that FDG-PET/MRI may be superior to FDG-PET/CT for the early radiographic evaluation of cervical cancers, with at least comparable diagnostic ability for detecting primary cervical and endometrial tumors and regional metastases [187]. Kirchner et al. (2017) reported sensitivity and specificity values of 50% and 58% for FDG-PET/MRI and 98% and 83% for FDG-PET/CT in diagnosing gynecologic cancers [188]. FDG PET/MRI appears to be a promising diagnostic modality for primary tumors, nodal staging, and recurrence in patients with pelvic gynecologic malignancies.

A systematic literature review by Virarkar et al. in 2020 evaluated the diagnostic performance of FDG PET/MRI for gynecological cancers, reporting sensitivity, specificity, DOR, and AUC values of 74.2%, 89.8%, 26, and 0.834, respectively [191]. Another meta-analysis by Virarkar et al. in 2020 compared the diagnostic performance of FDG PET/CT versus FDG PET/MRI for gynecological pelvic malignancies. This review of nine studies published up to 2019 found sensitivity and specificity values of 62.6% and 91.6% for PET/CT and 73.3% and 91.2% for PET/MRI, respectively, with no significant difference in DOR between the two modalities [192]. Compared with PET/CT, PET/MRI seems to have slightly better diagnostic performance than FDG PET/CT in gynecological malignancies. It is essential to note that the evaluation of the female pelvic region using FDG-PET can be affected by various factors, including the menstrual cycle and menopausal status. Incidental findings in the female genital region can also pose diagnostic challenges due to potential false positives and false negatives, particularly in cases of infections and post-therapy changes [193].

##### Detection and Prediction of Metastases of Gynecological Cancers

We also assessed the diagnostic value of FDG PET/CT in detecting and predicting metastases in gynecological cancers through four studies [190,194,195,196,197]. Jónsdóttir et al. reported sensitivity and specificity values of 43.5% to 100% and 50% to 100%, respectively, for PET/MRI in detecting and predicting metastases in the small bowel regions [197]. Gee et al. found that PET/CT demonstrated sensitivity and specificity values of 54.8% and 97.7% for CC metastasis and 64.6% and 98.6% for EC metastasis, respectively [194]. Kitajima et al. reported sensitivity, specificity, and accuracy values of 91.3%, 100%, and 93.3%, respectively, for PET/CT in detecting intra-pelvic recurrence/metastasis [190]. However, Bentivegna et al. observed a low accuracy of FDG PET/CT in predicting pelvic nodal status for patients with early-stage cervical and vaginal cancer [196]. A systematic review by Liu et al. in 2023, which included 13 studies with 473 patients and 2775 lesions, calculated detection rates for FDG PET/CT in the primary staging and recurrence assessment of pelvic cancers. The detection rate for early-stage assessment was 0.91, while it was 0.56 for the recurrence assessment of pelvic cancer. The sensitivity and specificity for detecting distant or lymph node metastases were 0.525 and 0.821, respectively [198]. In summary, FDG PET/CT and FDG-PET/MRI show promise in diagnosing primary and recurrent gynecological cancers, with some variations in sensitivity and specificity. These modalities also have potential in detecting metastases in pelvic cancers, although diagnostic challenges should be considered. Further research is needed to fully understand the diagnostic capabilities and nuances of these imaging techniques in the management of gynecological cancers. The domains of FDG PET/CT and FDG-PET/MRI in the management of gynecological cancers are summarized in Table 5.

##### Limitation and Strengths

The primary limitation of this study arises from the diverse methods and patient characteristics reported in the included studies, resulting in substantial heterogeneity in sensitivity, specificity, and diagnostic accuracy estimates.

Nonetheless, despite this limitation, the comprehensive review of published studies involving FDG PET/CT and FDG-PET/MRI methods still provides valuable insights into their roles in the management of gynecological cancers.

## 4. Conclusions

The early detection of gynecological malignancies plays a pivotal role in patient management and can significantly improve survival rates. This article offers an updated review of all the relevant studies on the application of FDG PET/CT and FDG PET/MRI in gynecological malignancies. Since 2004, FDG PET/CT has been increasingly employed for various purposes, including tumor staging, primary tumor diagnosis, recurrence prediction, metastasis detection, treatment prognosis assessment, and prognostic value prediction in gynecological malignancies. Furthermore, PET/MRI, as a promising “one-stop shop” approach, has been explored in patients with different gynecologic malignancies since 2008. These studies have evaluated the role of PET/MRI in staging, primary tumor diagnosis, and recurrence and metastasis detection in gynecologic malignancies. According to the available literature, FDG PET/MRI shows promise in enhancing cancer staging. However, its potential impact on prognosis, clinical decision-making, and treatment strategies for gynecological cancers remains to be fully determined. The development of rapid acquisition protocols and optimized attenuation correction methods is essential in this regard.

Despite the numerous advantages of PET/MRI and PET/CT in managing gynecological cancers, they come with challenges. These include limited capability to detect early-stage or small tumors, a risk of high false-negative results, and relatively low specificity [199]. Additionally, distinguishing tumors from benign lesions, such as corpus luteum cysts, dermoid cysts, and serous cysts, remains a challenge for these imaging modalities [199,200,201].

Another hurdle to consider is the cost-effectiveness of these imaging methods. Given the higher equipment cost, longer examination time, and expertise required, the incremental cost of PET/MRI is currently estimated to be as high as USD 4000 per examination. Nonetheless, the use of these modalities should be justified by their potential to improve the survival rates of patients with gynecological cancers, outweighing the additional costs and the exposure to ionizing radiation associated with their use. Consequently, numerous prospective studies employing standardized methods are needed before hybrid molecular imaging can be established as the primary imaging approach for gynecological cancers.

## Figures and Tables

**Table 1:** FDG PET/CT and FDG-PET/MRI in management of endometrial cancer (EC).

Domain	Imaging	References		Sensitivity		Specificity	Accuracy
Staging or diagnosis of primary EC	FDG PET/CT	[7,9,12,13,14,15]	FDG PET/CT	Staging	90–93%	49–96.3%	77.2%,
				Diagnosis	90.6–98.1%	33.3–99%	80–97%
	FDG-PET/MRI	[8]	FDG-PET/MRI	Staging	77%	84%	86.0%
	FDG-PET/MRI vs. FDG PET/CT	[10,11]		Diagnosis	-	-	-
Diagnosis or prediction of recurrence of EC	FDG PET/CT	[17,18,19,20,21]	FDG PET/CT		88.9%	93.6%	91–97%
Detection or prediction of metastases of EC	FDG PET/CT	[23,24,25,26,27,28,29,30,31,32,33,34,35,37]	FDG PET/CT		45.4–100%	66.67–99.8%	69.23–99.2%
	FDG-PET/MRI	[38]	FDG-PET/MRI		100%	96.9%	81.8–97%
	FDG PET/MRI vs. FDG PET/CT	[36]					

**Table 2:** FDG PET/CT and FDG-PET/MRI in management of ovarian cancer (OC).

Domain	Imaging	References		Sensitivity	Specificity	Accuracy
Staging and diagnosis performance of OC	FDG PET/CT	[41,43,45,46,47,48,49,50,51,52,105]	Staging	78%	68%	71%
			Diagnosis	82.4–94.7%	76.9–100%	81.1–92.9%
	FDG-PET/MRI	[44]	Staging	-	-	92.5%
			Diagnosis	-	-	
Predict optimal primary treatment/treatment prognosis and response of OC	FDG PET/CT	[55,56,57,58,59,60]		91%	67%	86%
Diagnosis or prediction of recurrence of OC	FDG PET/CT	[61,62,63,64,65,66,67,68,69,70,71,72,73,74,75,76,77,78,79,80,81,82,83,84,85,86,87,88,89,90,91]		40.7–100%	55.5–100%	72–97%
Detection or prediction of metastases of OC	FDG PET/CT	[42,93,94,95,96,97,98,99,100]		64–96.2%	90–98.2%	88.6–95.6%
Prediction of OC survival	FDG PET/CT	[103,104]		-	-	-

**Table 3:** FDG PET/CT and FDG-PET/MRI in management of cervical cancer (CC).

Domain	Imaging	References		Sensitivity		Specificity	Accuracy
Staging or diagnosis of primary CC	FDG PET/CT	[107,108,109,112,113,114,115,117,118]	FDG PET/CT	Staging	0–53.5%	81.87–100%	96%
				Diagnosis	67–86.66%	44.44–91%	76.92–95.45%
	FDG-PET/MRI	[110,111]	FDG-PET/MRI	Staging	77–91%	90–94%	87–93%
	FDG-PET/MRI vs. FDG PET/CT	[106]		Diagnosis	64.9%	-	78.5%
Treatment prognosis and prediction of response to treatment of CC	FDG PET/CT	[122,123,124,125,126,127,128,129]			20–94.8%	62–100%	89%
Diagnosis or prediction of recurrence of CC	FDG PET/CT	[132,133,134]			90.3%	81.0%	86.5%
Detection or prediction of CC metastasis	FDG PET/CT	[136,137,138,139,140,141,142,143,144,145,146,147,148,149,150,151,152,153,154,155,156]			28.6–92.8%	58.33–98.8%	65.1–99.3%
Prognostic value and prediction of survival of CC	FDG PET/CT	[160,161,162,163,164,165]			-	-	-

**Table 4:** FDG PET/CT in management of vulvar and vagina cancers.

Domain	Imaging	References		Sensitivity	Specificity	Accuracy
Staging or diagnosis performance of vulvar and vaginal cancer	FDG PET/CT	[168,169,170,171,172,173,174,175]	Staging of vulvar cancer	50–100%	65.5–89%	59–71.6%
			Diagnosis	95–100%	-	-
Recurrence of vulvar cancer	FDG PET/CT	[177]		100%	92%	98%
Detection and prediction of metastases vulvar and vaginal cancer	FDG PET/CT	[178,179,180]		56–89%	67–88%	74–84%

**Table 5:** FDG PET/CT and FDG-PET/MRI in management of gynecological cancers.

Domain	Imaging	References
Diagnosis of primary or recurrent GYN cancers	FDG PET/CT	[181,182,183,184]
	FDG PET/MRI	[185,186]
	FDG PET/CT vs. FDG PET/MRI	[187,188,189,190]
Detection and prediction of metastases GYN cancers	FDG PET/CT	[194,195,196]
	FDG PET/MRI	[197]

## Data Availability

All data generated or analyzed during this study are included in this published article.
